# The Behavior of Organic Phosphorus under Non-Point Source Wastewater in the Presence of Phototrophic Periphyton

**DOI:** 10.1371/journal.pone.0085910

**Published:** 2014-01-21

**Authors:** Haiying Lu, Linzhang Yang, Shanqing Zhang, Yonghong Wu

**Affiliations:** 1 State Key Laboratory of Soil and Sustainable Agriculture, Institute of Soil Science, Chinese Academy of Sciences, Nanjing, P. R. China; 2 Jiangsu Academy of Agriculture Sciences, Nanjing, P. R. China; 3 Centre for Clean Environment and Energy, Environmental Futures Centre, Griffith School of Environment, Gold Coast Campus, Griffith University, Queensland, Australia; Federal University of Rio de Janeiro, Brazil

## Abstract

To understand the role of ubiquitous phototrophic periphyton in aquatic ecosystem on the biogeochemical cycling of organic phosphorus, the conversion and removal kinetic characteristics of organic phosphorus (Porg) such as adenosine triphosphate (ATP) were investigated in the presence of the periphyton cultured in artificial non-point source wastewater. The preliminary results showed that the periphyton was very powerful in converting P_org_ evidenced by the fact that inorganic phosphorus (P_inorg_) content in solution increased from about 0.7 to 14.3 mg P L^−1^ in 48 hours in the presence of 0.6 g L^−1^ periphyton. This was because the periphyton could produce abundant phosphatases that benefited the conversion of P_org_ to P_inrog_. Moreover, this conversion process was described more suitable by the pseudo-first-order kinetic model. The periphyton was also effective in removing P_org_, which showed that the P_org_ can be completely removed even when the initial P_org_ concentration was as high as 13 mg P L^−1^ in 48 hours in the presence of 1.6 g L^−1^ periphyton. Furthermore, it was found that biosorption dominated the P_org_ removal process and exhibited the characteristics of physical adsorption. However, this biosorption process by the periphyton was significantly influenced by biomass (absorbent dosage) and temperature. This work provides insights into P_org_ biogeochemical circulation of aquatic ecosystem that contained the periphyton or similar microbial aggregates.

## Introduction

The discharge of excessive phosphorus into isolated water bodies will accelerate the eutrophication process. These water bodies such as lakes and dams suffer from severe water quality problems that are closely linked with the excessive phosphorus inputs from various pollution sources. This happens in both developed and developing countries [1], [2]. Thus, the removal measures of phosphorus from these water bodies, especially biological methods based on biofilms, have currently been a subject of great concern [3].

Organic phosphorus (P_org_) commonly includes nucleic acids, phospholipids, inositol phosphates, phosphoamides, phosphoproteins, sugar phosphates, amino phosphoric acids and organic condensed phosphorus species. It is often at least as abundant as (sometimes great excess of) inorganic phosphorus (P_inorg_) in natural water bodies and sediments [4]. Previous studies show that soluble P_org_ in water system, especially in lakes, often exceeded that of orthophosphate and accounted for 50%–90% of total phosphorus [5], [6]. In aquatic systems, the role of P_org_ has largely been underestimated not only because of its complexity in composition and structure [7], but also due to the limitation in analytical methods and techniques. As a result, P_org_ has usually been grouped in the “non-reactive” and “non-bioavailable” component of total phosphorus (P_total_) [4]. However, there is strong evidence that some organisms such as algae and bacteria are adapted to Porg via enzymatic hydrolysis and/or bacterial decomposition [8]–[10]. As a result, the importance of P_org_ is not widely recognized as a potentially large pool of bioavailable phosphorus, and its influences on phosphorus cycling and eutrophication of aquatic ecosystem are inevitably ignored among organic phosphorus species. Most importantly, P_org_ is typically not susceptible to the traditional removal technologies for the inorganic phosphorus [11], which may be due to its complicated species and chemical dynamics.

Phototrophic periphyton is mainly composed of multilayered consortia of photoautotrophs (e.g., cyanobacteria and microalgae) and heterotrophs (e.g. bacteria, fungi and protozoa), which is dominated by photoautotrophic microorganisms. These multilayer constructions are embedded in a common extracellular polymeric substance (EPS), secreted by the community, which mediates the adhesion of phototrophs and heterotrophs as well as gas and nutrient fluxes [12]. The periphyton is ubiquitous in aquatic environments and performs numerous important environmental functions such as nutrients cycling and self-purification of aquatic ecosystems [13], [14]. It has been proven that the periphyton has a high affinity for inorganic phosphorus and can act as an important potential sink for phosphorus in wetlands [15]. Thus, the periphyton has subsequently been developed to remove inorganic phosphorus from wastewaters due to its cost-effectiveness, easy-harvesting, high-effectiveness and environment-friendly advantages [16]. However, information about organic phosphorus utilization and removal by the periphyton such as kinetic analysis is still very limited. Although ATP represented by organic phosphorus had been investigated [17], the detailed removal mechanism and transformation as well as removal kinetics are still not clear.

Also, most current studies about phosphorus removal methods or technologies were focused on purely inorganic phosphorus or a specific type of phosphorus-based contaminants such as organophosphorus pesticides [18]. Moreover, it was postulated previously that the periphyton was capable of transforming P_org_ to P_inorg_ because of high phosphatase activities [19], which could introduce confusion between removal and conversion of P_org_ due to that P_org_ is traditonally calculated as the difference between P_total_ and P_inorg_. Therefore, a comprehensive investigation on P_org_ conversion and removal process by the periphyton ubiquitously distributed in natural waters will not only develop a potential technology for P_org_ removal from high-organic waters such as animal wastes, but also provide strong evidence to fully understand the phosphorus biogeochemical cycling of aquatic ecosystem that contain the periphyton or similar microbial aggregates.

In this work, we attempt to remove P_org_ from non-point source wastewater using the periphyton. The main objectives of this study were to (i) quantify the conversion and removal kinetic processes of P_org_ in the presence of the periphyton; (ii) evaluate the removal mechanism of P_org_ by the periphyton; (iii) explore the influence of environmental conditions to P_org_ removal by the periphyton.

## Materials and Methods

### Ethics statement

The study was not involved in any endangered or protected species. The investigation was permitted by Xuanwu Hu lake Management Committee, which is a public and benefit organ.

### Phototrophic periphyton culture

The biofilm substrate - Industrial Soft Carriers (Diameter 12 cm and length 55 cm, Jineng environmental protection company of Yixing, China) was used for *in situ* collecting and culturing of periphyton biofilms from Xuanwu Lake, East China. During the experiment, the substrates were submerged into the lake water (total nitrogen: 1.90 mg L^−1^, total phosphorus: 0.1 mg L^−1^, pH: 7.8, ammonia: 0.53 mg L^−1^; nitrate: 0.73 mg L^−1^); and the microorganisms in the hypereutrophic water as inoculums attached on the substrate surfaces and formed periphyton biofilms. Once the biofilms was covered on the substrate surface, the periphytons with their substrates were taken out for indoor culture.

The indoor culture of the periphyton was conducted in glass tanks (each tank: 100 cm length, 100 cm width, and 60 cm height). Firstly, the tanks were sterilized using a 95% alcohol solution and rinsed with water. Then, the collected periphyton along with their substrates were submerged into the glass tanks filled with simulated artificial wastewater [composed of macro nutrient (20 mg L^−1^ NaCO_3_, 150 mg L^−1^ NaNO_3_, 40 mg L^−1^K_2_HPO_4_, 75 mg L^−1^ MgSO_4_·7H_2_O, 36 mg L^−1^ CaCl_2_·2H_2_O) and micro nutrient (2.86 mg L^−1^ H_3_BO_4_, 1.81 mg L^−1^ MnCl_2_·4H_2_O, 0.22 mg L^−1^ ZnSO_4_, 0.39 mg L^−1^ Na_2_MoO_4_, 0.079 mg L^−1^ CuSO_4_·5H_2_O, 4.94 mg L^−1^ Co(NO_3_)_2_·6H_2_O) as well as organic matters (6 mg L^−1^ citric acid and ammonium ferric citrate)]. To avoid the influence of climatic condition on the periphyton growth, the glass tanks were kept in a greenhouse with air temperature at 25–30°C. When dense the periphyton was formed (the thickness of the periphyton exceeded 5 mm) after 60 days, it was collected for the following experiments.

### Characterization of the periphyton

The morphology of the periphyton was observed with Optical Microscopy (OM), Scanning Electron Microscope (SEM) and Zeiss Confocal Laser Scanning Microscope (CLSM). The microbial diversity of phototrophic periphyton was investigated using the method of Biolog™ ECO Microplates [20]. Briefly, 1 g of the periphyton (wet weight) were peeled off and cleaned under sterile conditions, and 150 µL aliquots were added into each well of every Biolog™ ECO Microplate, which was incubated at 25°C and color development (590 nm) was evaluated using a Biolog Microplate Reader every 12 h for seven days (168 h). Based on the pre-experiment, the ratio of dry to wet weight of the periphyton is 0.0532±0.0085 (average ± SD, n = 10), then 5% was selected as standards for dry weight calculation in all experiments subsequently.

### P_org_ stock preparation

Previous studies found that ATP was an effective substrate for tracing organic phosphorus dynamics in phytoplankton and periphyton [17], [21]. Thus, P_org_ stock solution (100 mg P L^−1^) was prepared by dissolving 0.6505 g ATP (disodium adenosine triphosphate, C_10_H_14_O_13_N_5_P_3_Na_2_·3H_2_O, sigma) into 1 L distilled water. All P_org_ concentrations used in experiments were diluted with the ATP stock.

### P_org_ conversion experiment

The conversion kinetic experiments of P_org_ were conducted in 250-mL flasks that contained 0 (control), 0.05, 0.1, and 0.2 g of the periphyton biomass in an incubator with an initial P_org_ concentration (C_0_) of about 20 mg P L^−1^ (conditions: light intensity  = 12000 Lux, temperature  = 25°C). The total phosphorus (P_total_) and inorganic phosphorus (P_inorg_) concentrations in solution were determined after 1, 4, 8, 12, 24, 36, and 48 hours, respectively.

To identify whether the phosphatase responsible for P_org_ conversion process in the presence of the periphyton, the method based on substrate para-nitrophenyl phosphate (pNPP) was used for phosphatase activities determination of the periphyton.

### P_org_ removal experiment

Five different treatment levels, each with different biomass (0, 0.1, 0.2, 0.4, 0.6 g) of the periphyton, were tested in this work. The periphyton was placed into 250-ml flasks with the artificial non-point source wastewater (without P_inorg_) of an initial P_org_ concentration at about 13 mg P L^−1^. P_total_ and P_inorg_ concentrations in solution were determined in 48 hours. To distinguish which mechanisms (adsorption and assimilation) are responsible for P_org_ removal process, 0.25 g NaN_3_ was added in solution to inhibit microbial activity and then the phosphorus concentrations in solution were determined.

To further evaluate the removal process, batch kinetic assay was conducted in 250-mL flasks under different biomass of phototrophic periphyton (0.2, 0.4 and 0.6 g) and temperature (10, 20, and 30°C). P_total_ and P_inorg_ concentrations in solution were determined after 1, 4, 8, 12, 24, 36, and 48 hours.

### Sample Analytical Methods

Dry weight (DW) of the periphyton was determined by oven drying samples at 80°C for 72 h. The biomasses of the periphyton used in the whole study (if not explained) were dry weights (g). The morphology of the phototrophic periphyton was characterized by optical microscope (OM), scan electronic microscope (SEM) and confocal laser scanning microscope (CLSM). The phosphatase assay procedure broadly follows that of Ellwood et al [19], Briefly, the periphyton biomasses cultured in different P_org_ concentration (from 10 to 50 mg P L^−1^) were carefully prepared, divided into similar sized aliquots and placed into 15-mL tubes containing 9.5 ml of artificial non-point source wastewater (without N or P), while the control contained no phototrophic periphyton. The tubes were then placed in a shaking incubator at 25°C for 20 min before the addition of 0.5 ml substrate (final concentration of 0.25 mM). The samples were then incubated for 3 h, after which the assay reaction was terminated by the addition 0.5 ml of 0.5 M NaOH. Finally, the phosphatase was determined using the absorbance of 405 nm wavelength. Biomasses of the periphyton were removed from the solution, rinsed and dried and weighed to an accuracy of 1.0 mg. The P_total_ and P_inorg_ concentrations in solution were determined simultaneously using a Flow injection analyzer (SEAL AA3, German).

### Data analyses

Each experiment in this study was conducted in triplicate, and the mean results (± SD) are presented. Statistical analysis was performed using SPSS 19.0, and p<0.05 indicated statistical significance. All figures were derived using Origin 8.0 and Excel 2007.

For the Biolog trial, average well color develop

ment (AWCD) was calculated according to the following equation:
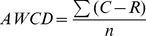
(1)where C is color production within each well, R is the absorbance value of the plate's control well, and n is the number of substrates (n = 31). Shannon index(H)is commonly used to characterize species diversity in a community, which was obtained by the following equation[22]:
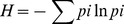
(2)where pi is the proportion of the relative absorbance value of well i to total plate's wells.

For enzyme activity assay, phosphatase activity (PA) was calculated by calibration curves constructed from p-Nitrophenol (pNP) standards (0 – 0.2 mM) in assay medium, which was expressed as mmol pNP released g^−1^ DW (dry weight) h^−1^ and obtained by the following equation:
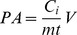
(3)where C_i_ is the concentration of pNP (mmol L^−1^), m is the dry weight of phototrophic periphyton (g), t is reaction time (h), and V is volume of solution.

For conversion kinetic study, the amount of P_org_ transformed to P_i_ (q_c_) at time t (0, 1, 2, 4, 8, 12, 24, and 48 h) was obtained based on Eq. 4:
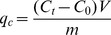
(4)where C_0_ is the initial P_inorg_ concentration (mg L^−1^), C_t_ is the concentration of P_inorg_ at time t, V is the volume of solution (L) and m is the dry weight of the periphyton (g).

Pseudo-first-order kinetic (Eq. 5) and Pseudo-second-order kinetic (Eq. 6) models were used to evaluate the conversion process [23]:
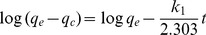
(5)where q_c_ and q_e_ represent the amount of P_org_ transformed to P_inorg_ (mg g^−1^) at time t and at equilibrium time, respectively. Parameter k_1_ represents the adsorption first-order rate constant (min^−1^) that calculated from the plot of log (q_e_−q_c_) against time (t).
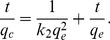
(6)where k_2_ is the pseudo-second-order rate constant (g mg^−1^ h^−1^). A plot between t/q_c_ versus t gives the value of the constants k_2_ and also q_e_ (mg g^−1^) can be calculated.

For P_org_ removal kinetic study, since there is no P_inorg_ in P_org_ solution, the amount of P_org_ removed (q_t_) at time t (0, 1, 2, 4, 8, 12, 24, and 48 h) was obtained based on Eq. 7:
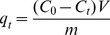
(7)where C_0_ is the initial total phosphorus concentration (mg L^−1^), C_t_ is the concentration of total phosphorus at time t, V is the volume of solution (L) and m is the dry weight of the periphyton (g).

Pseudo-first-order kinetic and Pseudo-second-order kinetic models (Eq. 5 and 6) were also used to evaluate the removal process. Moreover, adsorption models such as the intra-particle diffusion (Eq. 8) and Arrhenius equation (Eq. 9) were used to further analyze the removal process, which were calculated as follows:
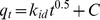
(8)where q_t_ is the amount removed at time t, parameter K_id_ (mg g^−1^ min^0.5^) is the rate constant of intra-particle diffusion.
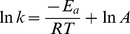
(9)where E_a_ is activation energy, T is the temperature in Kelvin, R is the gas constant (8.314 J mol^−1^ K^−1^) and A is a constant called the frequency factor. Value of E_a_ can be determined from the slope of ln k versus T^−1^ plot.

## Results and Discussion

### Characteristics of the phototrophic periphyton

**Figure 1:**
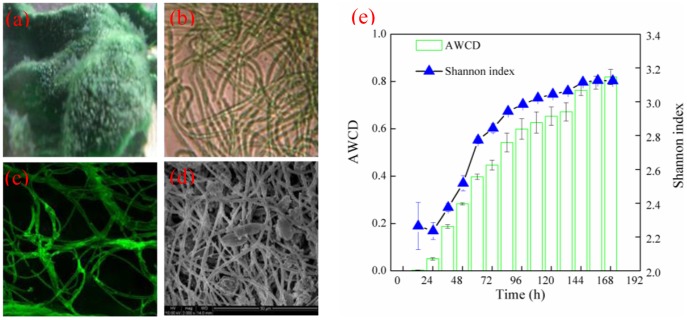
Characteristics of the the periphyton. The photo of the periphyton employed for the experiments (a), the periphyton observed under OM (b, ×2000), CLSM (c, ×2000), and SEM (d, ×2000); the microbial community diversities of the periphyton based on Biolog analyses (e).

It was observed that the periphyton was mainly composed of green algae, diatoms, bacteria, and protozoa, which was dominated by phototrophic algae (Fig.1). These algae with a diameter of about 1.5 um intertwined each other, forming the base matrix for other microorganisms such as bacteria. The Shannon index of the periphyton based on the Biolog analyses was about 3.1 after 7 days, indicating that there were many types of microorganisms living in the periphyton [24].

Micro-structure is a significant determinant in the activity of the biofilm since it plays an important role in the transportation of nutrients and waters [14]. Previous studies indicated biofilms structure was heterogeneous and complex, which contained voids, channels, cavities, pores, and filaments and with cells arranged in clusters or layers [25]. As shown in the CSLM image that the periphyton composed of biomass clusters separated by interstitial voids, which might have considerable consequences on mass transfer inside the biofilms and exchange of substrates and products with the water phase. Because such micro-voids of the periphyton could play many important roles such as interception in nutrients transportation, especially granular nutrients, between sediment and water interface. However, such structure characteristic was obviously influenced by the species arrangement of organisms that composing the biofilms [26]. For example, the micro-voids constructed among complex cells such as algae, bacteria and protozoa may be larger than these constructed by single species. These voids might also provide more micro-spaces or adsorption sites for capturing nutrients,especially for the particulate nutrients such as polyphosphate particles. It may assisted in the understanding of self-purification of aquatic systems that contain the periphyton [14].

### P_o_ conversion process by the periphyton

**Figure 2:**
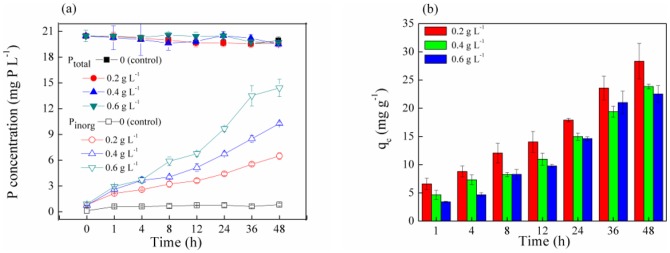
The conversion process of P_org_ (a) the change of the P_total_ and P_inorg_ over the time (b) the change of q_c_ over the time (experiment conditions: light intensity  = 12000 Lux, temperature  = 25°C).

The transformation process of P_org_ (ATP) by the periphyton was studied by monitoring the P_total_, P_inorg_ and q_c_ over time (Fig. 2). When the initial P_org_ concentration was about 20 mg P L^−1^, the P_inorg_ concentrations in solution were obviously increased over times from about 0.7 to 6.4, 10.2, and 14.3 mg P L^−1^ under 0.2, 0.4, and 0.6 g L^−1^ of the periphyton content respectively, while the control (no periphyton) showed no significant change in 48 h (*P*>0.05). These indicated that the periphyton had relatively substantial transformation ability to convert P_org_ to P_inorg_, which became stronger with the increasing biomass of the periphyton. It is well known that the reaction rate of (conversion rate of P_org_ to P_inorg_) was directly associated with phosphatase based on the reaction equation [ATP+enzyme → ADP+Pi+energy]. This means that the periphyton produced phosphates, which is beneficial to the P_org_ conversion reaction. Moreover, the content of phosphates increased with the increasing biomass of periphyton.

To quantify the transformation capacity of P_org_ by the periphyton, the P_org_ transformation data were described using kinetic models (Pseudo-first-order and Pseudo-second-order kinetic equation). According to Fig. 2b, the amounts of P_org_ converted to P_inorg_ (q_c_) after 48 h by the periphyton were 28.3, 23.9, and 22.5 mg g^−1^ under 0.2, 0.4, and 0.6 g L^−1^, respectively. The pseudo-first-order and pseudo-second-order kinetic constants k and q values determined from the plots, decreased with the enhancement in the biomass of the periphyton (Table 1). This implies that the treatment with higher periphyton biomass contains a dense layer, resulting in smaller contribution on P_org_ transformation. This result is consistent with the observation in previous studies [27], [28]. It is notable that the fitting correlation coefficient (R^2^) of pseudo-first-order model is better than that of the pseudo-second-order coefficient, suggesting that the pseudo-first-order kinetic model is more suitable for P_org_ transformation process.

**Table 1:** The kinetic parameters of P_org_ transformation by the periphyton.

Periphyton biofilm content	Pseudo-first-order kinetic model			Pseudo-two-order kinetic model		
	k_1_(h^−1^)	q_1_ (mg·g^−1^)	R^2^	k_2_ (g·mg^−1^·h^−1^)	q_2_ (mg·g^−1^)	R^2^
0.2 g L^−1^	0.0432	24.632	0.964	0.0028	31.847	0.922
0.4 g L^−1^	0.0416	21.463	0.977	0.0026	27.778	0.897
0.6 g L^−1^	0.0412	20.888	0.984	0.0017	30.303	0.888

**Figure 3:**
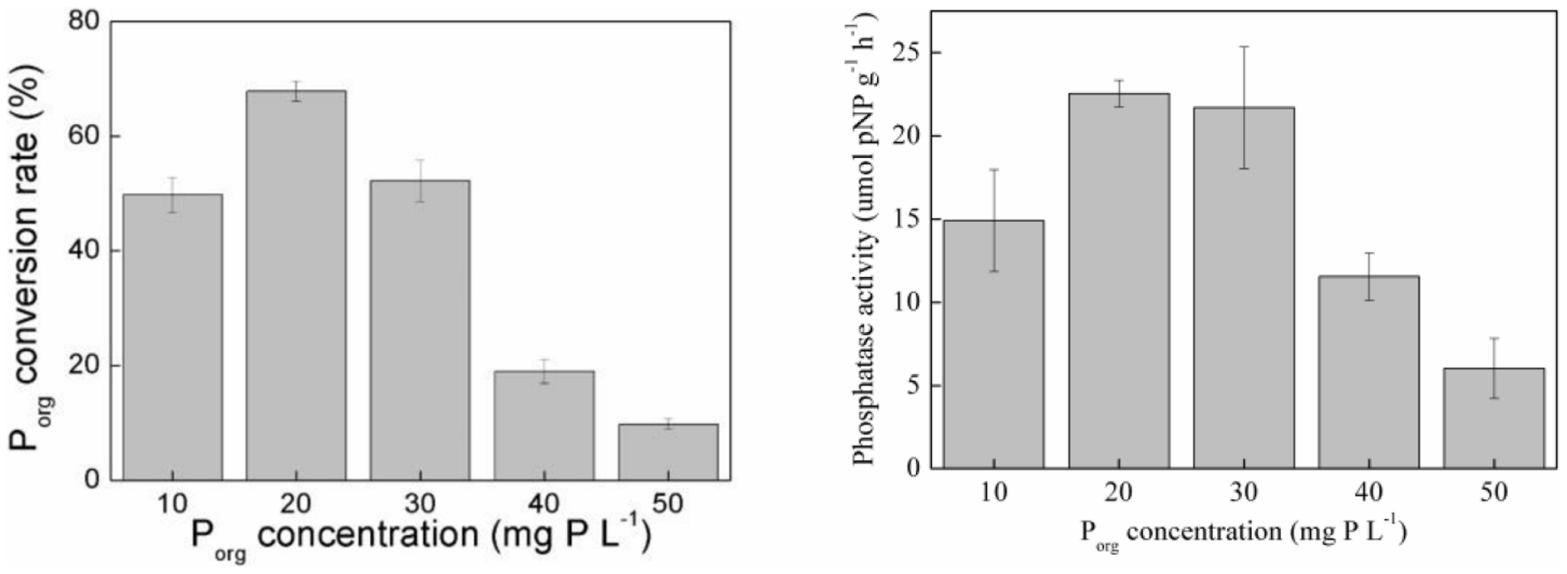
Phosphatase activity of the periphyton under different P_org_ concentrations.

To identify the role of phosphatase on P_org_ transformation by the periphyton, phosphatase activity under varied P_org_ concentration was evaluated. The phosphatase activity was increased initially but decreased subsequently as P_org_ concentration increased (Fig. 3). The maximal phosphatase activity was about 22 µmol pNP g^−1^ h^−1^ when the P_org_ concentration was 20 mg P L^−1^. Phosphatase plays an important role in the biochemical cycles of phosphorus in aquatic system by hydrolyzing dissolved organic phosphorus to phosphates that are available for cellular uptake. Such enzymatic response to phosphorus limitation has been demonstrated previously in both planktonic communities and biofilms [21], [29], [30]. Our experiment also showed similar phosphatase activities, which demonstrates that phosphatase was a main factor affecting P_org_ conversion process.

The phototrophic biofilm cultured under limited phosphate and organic phosphorus supply exhibited higher phosphatase activities than that cultured with sufficient phosphate supply [19], suggesting that end-product repression and de-repression of phosphatase activity was a main limitation factor of phosphatase activity. In this study, the P_inorg_ concentration transformed from P_org_ increased slowly when the periphyton mass was under 0.6 g L^−1^ in late time (after the 36 h) (Fig. 2).This result is similar with Ellwood's (2012) study. However, it was found that the P_org_ concentration also was an important factor determining the phosphatase activity of phototrophic periphyton (Fig. 3). There is a significant reduction in phosphatase activity under high P_org_ concentration (*p*<0.05). One possible explanation could be the growth repression under high P_org_ concentration, which showed that the periphyton were not functioning after being cultured in high P_org_ concentration (i.e., over 30 mg P L^−1^).

The conversion from P_org_ to P_inorg_ is an important process for phosphorus removal and recovery since P_inorg_ is the removable and recoverable form of phosphorus in wastewater-treatment system. Many measures such as the advanced-oxidation processes (AOPs) have been applied for converting P_org_ to P_inorg_, which are regarded as promising means for the transformation of P_org_ in the low-concentration streams [11]. AOPs rely on non-specific free-radical species, such as hydroxyl radicals, to quickly attack the structure of organic compounds. However, this method mostly applied for the destruction of specific P-based and trace contaminants, such as organophosphorus pesticides [31]. Therefore, the AOPs methods might be impractical to apply in surface waters such as stream and lake, which often have high organic pollution loadings such as animal discharge. Compare to AOPs methods, many advantages such as low capital cost, easy-harvest, and powerfully converting ability for high content and non-specific P_org_ (ATP), suggesting that periphyton-based conversion system is a potential promising technology for P_org_ removal and recovery.

### P_org_ removal by the periphyton

**Figure 4:**
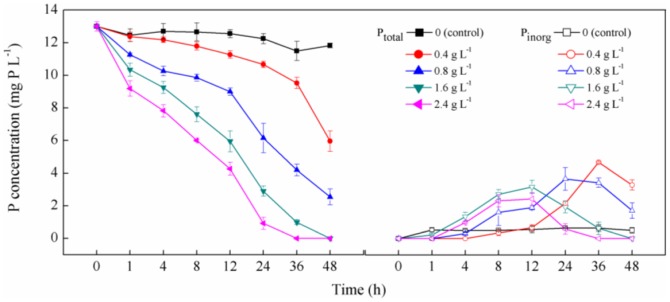
The removal process of P_org_ by the periphyton. P_total_ means total phosphorus content and P_inorg_ means inorganic phosphorus content.

The P_total_ concentration in solution was decreased over time from 13 mg P L^−1^ to 6, 2, 0, and 0 mg P L^−1^ respectively under the treatments with the periphyton masses of 0.4, 0.8, 1.6, and 2.4 g L^−1^ respectively while the P_total_ content in the control showed slight reduction (Fig. 4). This indicates that the periphyton could remove P_org_ from artificial non-point source wastewater effectively. Simultaneously, the P_inorg_ concentration in solution showed the same change under varied biomass content treatments, which was firstly increased and then decreased. This indicates that the conversion of P_org_ by the periphyton occurred throughout the whole removal process. However, according to the P_org_ conversion trial (Fig. 2a), the concentrations of P_inorg_ transformed from P_org_ in solution should increase continuously over times and are larger than the P_inorg_ contents determined in Fig. 4. This implies that removing P_inorg_ that converted from P_org_ is a key procedure in the P_org_ removal process by the periphytons.

**Figure 5:**
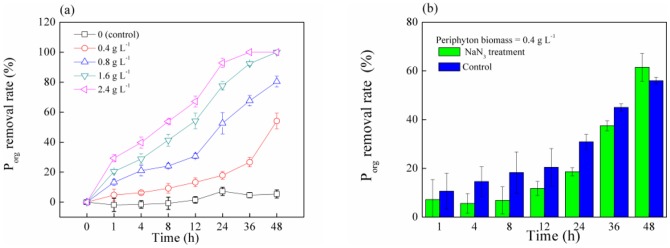
The P_org_ removal rate under different treatments.

According to Fig. 5a, the removal rate under the four periphyton biomass levels (0.4, 0.8, 1.6, and 2.4 g L^−1^) demonstrated a consistent trend over time within 48 h, and increased from 5%, 13%, 20%, and 29% to 54%, 80%, 100%, and 100%, respectively. This implies that the higher the content of the periphyton is available for P_org_ removal, the greater the amount of P_org_ is removed. Furthermore, to determine whether assimilation mechanism dominated the removal process of P_org_ by the periphyton, NaN_3_ was used to impede microbial activity of the periphyton by restraining microbial respiration and inhibiting assimilation [32], [33]. The removal rates of P_org_ by the periphyton under NaN_3_ treatment within 48 h were not significantly different from the controls (*p*>0.05, Fig. 5b), which indicates that the assimilation of phosphorus by microbes was minimal during the removing of P_org_ by the periphyton in 48 h. This further suggests that the P_org_ removal process of the periphyton is dominated by adsorption within 48 h.

**Figure 6:**
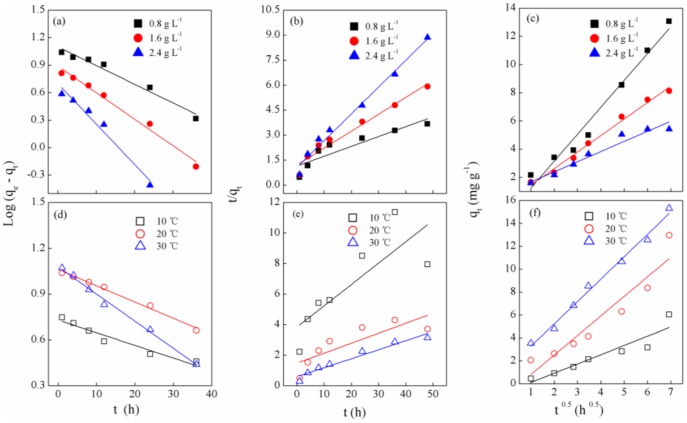
Adsorption kinetic analysis, (a d) the pseudo first-order kinetic and (b e) the pseudo second-order kinetic and (c f) the intra-particle diffusion kinetic of the periphyton biofilm for the P_org_ with different biomass content at different temperatures.

For a pseudo-second-order model, the correlation coefficient (R^2^) is generally less than the pseudo-first-order coefficient (Table 2). Accordingly, kinetic parameters k_1_ and q_1_ showed the same trend that increased from 0.019 and 5.84 to 0.041and 11.97 respectively with temperature rise, while k_1_ increased from 0.047 to 0.102 and q_1_ decreased from 12.57 to 4.99 with the periphyton content increased. In view of these results, it can be safely concluded that the pseudo-first-order kinetic model provides a better correlation and description for the adsorption process of P_org_ by the periphyton at different temperatures and biomass (Fig. 6).

**Table 2:** Kinetic parameters for P_org_ removal by the periphyton.

Treatments	Pseudo-first-order kinetic			Pseudo-two-order kinetic			Intra-particle diffusion		
	k_1_ (h^−1^)	q_1_ (mg·g^−1^)	R^2^	k_2_ (g·mg^−1^·h^−1^)	q_2_ (mg·g^−1^)	R^2^	k_id_ (mg·g^−1^·h^−0.5^)	C	R^2^
10°C	0.019	5.84	0.944	0.005	7.15	0.670	0.810	−0.71	0.876
20°C	0.025	11.51	0.991	0.003	15.34	0.707	1.707	−0.91	0.901
30°C	0.041	11.97	0.994	0.006	16.95	0.953	1.955	1.31	0.991
0.8 g L^−1^	0.047	12.57	0.972	0.003	16.86	0.843	1.908	−0.66	0.973
1.6 g L^−1^	0.067	7.74	0.986	0.009	9.85	0.961	1.173	0.27	0.990
2.4 g L^−1^	0.102	4.99	0.973	0.024	6.22	0.966	0.719	0.95	0.953

In a solid-liquid system, most adsorption reactions take place through multi-step mechanisms, which at least contain external film diffusion, intra-particle diffusion, and interaction between adsorbate and active site. Thus, an intra-particle diffusion model was chosen to analyze the process of P_org_ adsorption onto the periphyton. The determination coefficients (R^2^) were increased from 0.88 to 0.99 as temperature rose (Table 2), which suggested intra-particle diffusion may be rate controlled step under high temperature (30°C). The relatively high R^2^ under different biomass contents (Table 2) implies that intra-particle diffusion in adsorption process of P_org_ by the periphyton was influenced by biomass. According to intra-particle diffusion model, if the plot of q_t_ versus t^0.5^ presents a multi-linearity correlation, it indicates that three steps occur during the adsorption process: the first is the transport of molecules from the bulk solution to the adsorbent external surface by diffusion through the boundary layer (film diffusion). The second portion is the diffusion of the molecules from the external surface into the pores of the adsorbent. The third portion is the final equilibrium stage, where the molecules are adsorbed on the active sites on the internal surface of the pores and the intra-particle diffusion starts to slow down due to the solute concentration becoming lower [34], [35]. It was shown that the plot of q_t_ versus t^0.5^ presents a multi-linearity correlation and does not pass through the origin under low temperature (Fig. 6f), which indicates the adsorption of P_org_ by the periphyton was control by some other processes than intra-particle diffusion process under relatively low temperature. The large intercept (C) suggests that the process is largely of surface adsorption. This implies that the adsorption of P_org_ by the periphyton at temperature of 30°C and biomass of 2.4 g L^−1^ were more inclined to surface adsorption (Table 2).

To further reveal the types of P_org_ adsorption (physical and chemical) by the periphyton, Arrhenius equation was chosen to calculate the activation energy (E_a_) based on kinetic parameters. The magnitude of E_a_ may give an idea about the type of adsorption. Two main types of adsorption may occur, physical and chemical. In physical adsorption, E_a_ value is usually low between 5–40 kJ mol^−1^ since the equilibrium is usually rapidly attained and the energy requirements are weak [36]. Chemical adsorption is specific and involves forces much stronger than physical adsorption, where E_a_ value is commonly high between 40 and 800 kJ mol^−1^ according to Arrhenius equation [37]. However, in some systems the chemical adsorption occurs very rapidly and E_a_ was relatively low, which is termed as a non-activated chemisorption [38].

The correlation coefficient of corresponding linear plot of ln k against 1/T is 0.96. The E_a_ value for the adsorption of P_org_ onto the periphyton is found to be as 27.082 kJ mol^−1^, which suggests that the adsorption of P_org_ in the presence of the periphyton is exhibited the characteristic of physical adsorption.

Organic phosphorus can be found commonly in municipal, agricultural, and animal wastewaters, but there is scant information on its removal and recovery due to current phosphorus removal techniques are typically for inorganic phosphorus. Furthermore, as phosphorus resources becomes more scarce recently, phosphorus recovery from wastewaters by algal and macrophyte are regarded as a promising strategy and already in widespread use [39]. Compared to algal and macrophyte, the periphyton are more easy to be acquired and harvested. Therefore, the development of removing and capturing phosphorus from non-point source wastewaters for reuse based on the periphyton are primarily important to agriculture in the near future. In this study, our experimental results reveal that the periphyton not only possesses substantial capacity in effective organic phosphorus removal, but also the great ability in converting organic phosphorus to inorganic phosphorus that are readily captured. Finally, there are many advantages of the periphyton itself - it is environmentally friendly, economically viable and operationally simple. Given the above advantages, this phosphorus removal, recovery and reusing technologies based on the periphyton will have vast practical potentials, although it is also dependent on numerous factors such as light, temperature, water column phosphorus concentration, water flow velocity, the growth stage and thickness of periphyton [40], [41]. Most importantly, the native conditions of wastewaters in natural system (especially in agricultural wastewaters) are more complicated and the adsorption process may be reversible by the periphyton under high flow conditions. In such conditions, whether the adsorbed phosphorus will be released into aquatic ecosystem later from the periphyton needs further investigation.

## Conclusions

Phototrophic periphyton can produce large amounts of phosphatase that can facilitate the transformation of organic phosphorus to inorganic phosphorus. This conversion process is influenced by the concentration of organic phosphorus and periphyton biomass in solution. Also, the periphyton can effectively remove organic phosphorus from artificial non-point source wastewaters, and the removal process is dominated by biosorption that exhibits the characteristic of physical adsorption. This bioadsorption process is distinct from the non-bioadsorption, which is more influenced by the environmental conditions such as temperature. This work gives an insight into the organic phosphorus conversion and removal processes in the presence of the periphyton or other similar microbial aggregates, contribute to the full understanding of phosphorus biogeochemical circulation in aquatic system, and provide kinetic data for the design of engineering of phosphorus removal, recovery and reusing technologies based on the periphyton.
